# Inhibitor treatment of peripheral mononuclear cells from Parkinson’s disease patients further validates LRRK2 dephosphorylation as a pharmacodynamic biomarker

**DOI:** 10.1038/srep31391

**Published:** 2016-08-09

**Authors:** G. Perera, M. Ranola, D. B. Rowe, G. M. Halliday, N. Dzamko

**Affiliations:** 1 Neuroscience Research Australia, Randwick, 2031, Australia; 2 Faculty of Medicine and Health Sciences, Macquarie University, Sydney, 2109, Australia; 3 School of Medical Sciences, University of NSW, Kensington, 2052, Australia

## Abstract

Activating mutations in leucine-rich repeat kinase 2 (LRRK2) are strongly associated with increased risk of Parkinson’s disease (PD). Thus, LRRK2 kinase inhibitors are in development as potential Parkinson’s disease therapeutics. A reduction in the constitutive levels of phosphorylation on leucine-rich repeat kinase 2 (LRRK2) is currently used to measure target engagement of LRRK2 kinase inhibitors in cell and animal models. We aimed to determine if reduced phosphorylation of LRRK2 following inhibitor treatment is also a valid measure of target engagement in peripheral mononuclear cells from Parkinson’s disease patients. Peripheral mononuclear cells from idiopathic Parkinson’s disease patients and controls were treated *ex vivo* with two structurally distinct inhibitors of LRRK2, at four different doses, and immunoblotting was used to assess the reduction in LRRK2 phosphorylation at Ser910, Ser935, Ser955 and Ser973. Both inhibitors showed no acute toxicity in primary cells and both inhibitors reduced the constitutive phosphorylation of LRRK2 at all measured residues equally in both control and Parkinson’s disease groups. Measuring the reduction in LRRK2 phosphorylation resulting from LRRK2 kinase inhibition, is thus a valid measure of acute peripheral target engagement in Parkinson’s disease patients. This is important if LRRK2 kinase inhibitors are to be used in a clinical setting.

Leucine-rich repeat kinase 2 (LRRK2) is a potential therapeutic target for the treatment of Parkinson’s disease (PD). Genome-wide association studies show an increased risk of idiopathic PD with certain *LRRK2* genetic variations^1^,^2^,^3^, whilst missense mutations in the catalytic core of the LRRK2 enzyme cause a familial form of PD that is largely indistinguishable from the idiopathic disease^4^,^5^,^6^. Although pathomechanisms remain to be fully elucidated, current evidence suggests a role for LRRK2 kinase activity in PD pathogenesis (for recent reviews see ref. 7, 8, 9). In particular, the most common pathogenic LRRK2 mutation, G2019S, occurs in the kinase domain and increases catalytic activity by 2-3 fold^10^. Consequently, substantial effort has gone into the development of potent and selective inhibitors of LRRK2 kinase activity^11^,^12^.

Key to the generation of LRRK2 kinase inhibitors was development of a cellular readout of LRRK2 kinase activity. The most widely used assay involves measuring phosphorylation levels on serine residues Ser910 and Ser935^13^, which are located prior to the leucine-rich repeat domain on the LRRK2 enzyme itself. Studies using cell culture, animal models and primary human cells have all demonstrated a dose-dependent reduction in the constitutive phosphorylation of LRRK2 Ser910 and Ser935 with increasing inhibitor concentration. Biologically, phosphorylation of both LRRK2 Ser910 and Ser935 is required for the binding of LRRK2 to 14-3-3 family adaptor proteins^14^. Loss of LRRK2 Ser910/Ser935 phosphorylation following inhibitor treatment results in disassociation of LRRK2 and 14-3-3, which at least in cell culture appears to alter the subcellular localization of LRRK2^13^,^15^. A dose-dependent reduction in the constitutive phosphorylation at two additional residues, Ser955 and Ser973, has also been shown following LRRK2 inhibitor treatment^16^, although these residues are not required for 14-3-3 binding and their biological role is currently unclear.

It is important to note however, that all four of these residues are not direct LRRK2 auto-phosphorylation sites. Rather, their phosphorylation is regulated indirectly by LRRK2 kinase activity, likely via intermediate signaling kinases and/or phosphatases^13^,^17^,^18^,^19^. This introduces a level of complexity into the pharmacodynamic assay and thus careful validation is required. This is particularly important if further translation of LRRK2 inhibitors from cellular studies and animal models to human clinical trials is warranted. Therefore, in the present study, we have investigated the dose-dependent decrease in the constitutive phosphosphorylation of LRRK2 Ser910, Ser935, Ser955 and Ser973 in primary human peripheral blood mononuclear cells from idiopathic PD patients and matched controls. We show using structurally distinct inhibitors of LRRK2 *ex vivo*, that the reduction of LRRK2 phosphorylation at all measured residues is similar between the PD and control groups. Moreover, LRRK2 inhibitors had no cytotoxic effects on primary immune cells. These results suggest that the reduction in LRRK2 phosphorylation seen with inhibitor treatment may be a viable peripheral pharmacodynamic biomarker for potential clinical trials of LRRK2 kinase inhibitors.

## Methods

### Viability and toxicology assays using PBMCs from healthy blood donors

Peripheral blood mononuclear cells (PBMCs) were isolated from buffy coat obtained from healthy volunteer blood donors to the Red Cross blood service by layering onto ficoll paque plus (GE healthcare) and centrifuging at 400 × g for 30 min. All studies with buffy coat were approved by the University of NSW human research ethics advisory panel (reference #HC14226) and the methods carried out in accordance with the approved guidelines and regulations. Cell count and viability by trypan blue exclusion was performed with an automated cell clounter (Countess, Life Technology) and 2 × 10^6^ cells were used for experiments. PBMC’s were then treated with LRRK2 inhibitors, dissolved in DMSO, PF06447475 (Sigma)^20^, GSK2578215A (Tocris)^21^, LRRK2-IN1(a kind gift from Dario Alessi)^15^ at 0.5 μm for 24 h. Untreated and DMSO alone treatments were included as controls. The release of lactate dehydrogenase in the tissue culture media was measured using a CytoTox 96^®^ Non-Radioactive Cytotoxicity Assay (Promega) according to the manufacturer’s protocol. ATP levels were measured using a CellTitre-Glo ATP assay (Promega), and plates for both ATP and LDH assays were read on a Polarstar Omega plate reader (BMG labtech). Apoptosis measurements were carried out according to the manufacturer’s protocol using an Annexin V/PI kit (Miltenyi Biotech). At least 20,000 events were acquired with a FacsCANTO II flow cytometer (BD Biosciences) and analysed using FlowJo version 10.0.7 (Treestar Inc.). Treated cell pellets were also collected for immunoblot analysis of total LRRK2 levels as described below.

### Subject recruitment

Subjects were recruited with informed consent and the study was approved by the Macquarie University Human Research Ethics Committee (reference 5201100874). Some additional control subjects were also recruited with informed consent through the Forefront program at Neuroscience Research Australia (approved by South Eastern Sydney Local Health District Human Research Ethics Committee, reference 10/92). All methods were performed in accordance with relevant guidelines and regulations. Clinic visits occurred between 9–11 am to minimize the impact of diurnal variation and subjects with less than 8 years from initial symptom onset were chosen. The control group predominantly comprised of unaffected spouses of PD subjects. Patients with a strong family history or early onset PD were excluded and no patient or control subject had an immune, inflammatory or neurological disorder other than PD. Clinical severity of PD was assessed by the Movement Disorders Society Unified Parkinson’s Disease Rating Scale (MDS-UDPRS)^22^. Use of dopamine replacement medication was recorded and levodopa equivalent dose calculated^23^. Demographic data are presented in Table 1.

### Isolation and treatment of PBMCs from participants

Blood from participants was collected into Vacutainer CPT cell preparation tubes with sodium heparin (BD Biosciences) and then centrifuged at 1600 × g for 20 min at room temperature in a swing bucket rotor (Multifuge 3SR, Thermofisher). The layer of peripheral blood mononuclear cells was removed by sterile Pasteur pipette and diluted with 10 volumes of RPMI-1640 medium before being pelleted by centrifugation at 300 × g for 7 min to reduce platelet contamination. The supernatant was discarded and PBMCs resuspended in 10 ml RPMI-1640 media for cell counting and viability measurements using trypan blue exclusion and an automated cell counter (Countess, Life Technology). 1 × 10^6^ cells were then treated with LRRK2 inhibitors GSK2578215A and LRRK2-IN1 at indicated concentrations for 1 hr. Cells were then centrifuged at 300 × g for 5 min and the pellets lysed in buffer consisting of 50 mM Tris.HCL pH 7.5, 1 mM EGTA, 1 mM EDTA, 1 mM sodium orthovanadate, 50 mM sodium fluoride, 5 mM sodium pyrophosphate, 0.27 M sucrose, 1 mM benzamidine, 1 mM PMSF and 1% (v/v) Triton X-100. The lysates were snap frozen and stored at −80 °C until analysis.

### Immunoblotting

Samples were thawed on ice and clarified by centrifuging at 10,000 × g for 20 min at 4 °C. Protein concentration was determined by bicinchoninic assay (Thermofisher Scientific) and samples made up in LDS sample buffer (Life Technologies). 10 μg of sample was separated with 4–12% Novex Tris-glycine gels (Life Technologies) and transferred onto nitrocellulose membrane (Biorad). Membranes were blocked with 5% skim milk powder in Tris buffered saline with 0.1% (v/v) Tween 20 (TBST). Membranes were probed overnight at 4 °C for LRRK2 (N241A/34, Neuromab) and phosphorylated LRRK2 P-Ser935 (UDD2, Abcam), P-Ser955 (MJF-R11, Abcam), P-Ser973 (MJF-R12, Abcam), P-Ser910 (UDD1, Abcam). All primary antibodies were used at 1:1000 dilution in 5% skim milk in TBST. All LRRK2 antibodies have been substantially validated for specificity using immunoblot^16^,^17^,^24^. After overnight incubation membranes were washed in TBST and then anti-rabbit and anti-mouse horseradish peroxidase (HRP) secondary antibodies (Biorad) were used at 1:5000 dilution in 2.5% skim milk in TBST. Enhanced chemiluminescence reagent (GE Healthcare) was used for detection using a Chemidoc MP Imaging system (Biorad). Immunoblot quantitation was performed using Imagelab software 5.1 (Biorad). Levels of phosphorylated LRRK2 were normalized to levels of total LRRK2 and expressed as a percentage of the untreated control PBMCs. For western blot analysis of total LRRK2 levels in healthy donor PBMCs, β-actin was used as a loading control. Representative cropped immunoblots are shown in the figures. Larger immunoblot examples are shown in Supplementary Figure 1.

### Statistical analysis

One-Way ANOVA with Dunnets post-hoc test was used to analyze the effect of different LRRK2 inhibitors on cell viability, toxicology and total protein levels of LRRK2. Two-way repeated measured ANOVA with Tukey’s posthoc test was used to analyze the effect of LRRK2 inhibitors on LRRK2 phosphorylation in the control and PD patient PBMCs. All analyses were performed using Prism software (Graphpad) with significance accepted at p < 0.05.

## Results

### LRRK2 inhibitors are not toxic to primary human immune cells

**Figure 1:**
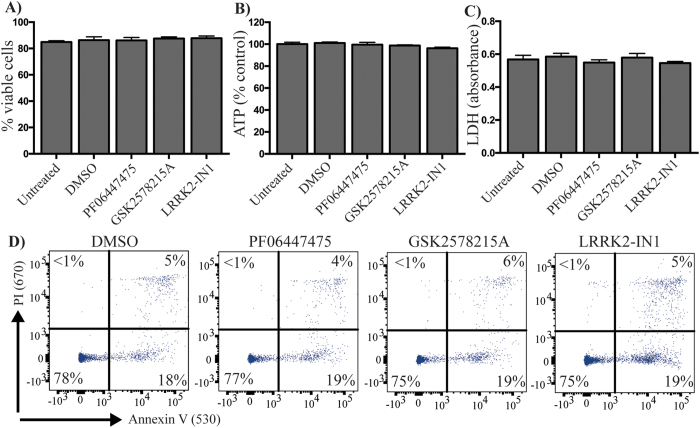
LRRK2 kinase inhibitors are not acutely toxic to primary human immune cells. Peripheral blood mononuclear cells were obtained from healthy blood donors to the Red Cross Blood Service. Mononuclear cells were isolated from buffy coat by ficol density centrifugation and treated with 0.5 μM of the indicated LRRK2 kinase inhibitors or DMSO as control for 24 h. Cell viability was assessed by trypan blue exclusion **(A)** and intracellular ATP levels **(B)**. The release of lactate dehydrogenase into the tissue culture media **(C)** was measured by absorbance assay. Annexin-PI staining and flow cytometry **(D)** were used to determine if LRRK2 kinase inhibitors were inducing apoptosis. Data are mean ± SEM. n = 4–6 biological replicates performed at least in triplicate.

We first assessed any acute toxicity of LRRK2 kinase inhibitors in primary human PBMCs obtained from healthy blood donors. Cells were incubated for 24 h with relatively high concentrations of inhibitors (0.5 μM). None of the three structurally distinct LRRK2 inhibitors investigated had any effect on cell viability as assessed by trypan blue exclusion (Fig. 1A) or cellular ATP levels (Fig. 1B). None of the inhibitors induced the extracellular release of lactate dehydrogenase (Fig. 1C) or the plasma membrane expression of annexin V (Fig. 1D), indicating no induction of necrosis or apoptosis. These results suggest that LRRK2 kinase inhibitors have no acute toxicity in primary human immune cells.

### LRRK2 inhibitors reduce LRRK2 expression in cultured PBMCs

**Figure 2:**
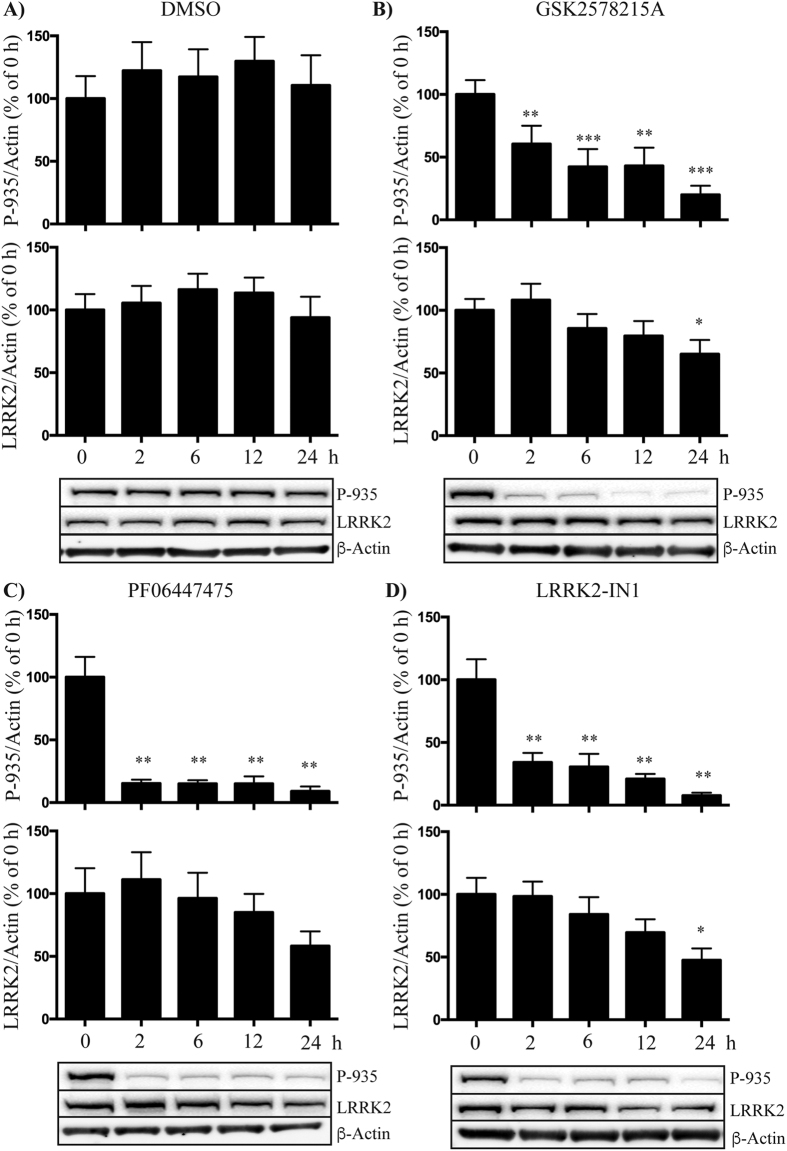
LRRK2 inhibitors may reduce total LRRK2 levels in primary human PBMCs. Peripheral blood mononuclear cells obtained from healthy blood donors were treated with DMSO **(A)** or 0.5 μM of GSK2578215A **(B)**, PF06447475 **(C)** or LRRK2-IN1 **(D)** over a 24 h time course. Samples were collected at indicated times for immunoblot analysis of LRRK2 phosphorylated at Ser935 and total LRRK2, which were corrected to β-actin following quantitation. A repeated measure ANOVA with Dunnet’s post hoc test was used to assess differences in total and Ser935 phosphorylated LRRK2 levels over time with significance accepted at p < 0.05. Results are expressed as the percentage of LRRK2 expression relative to the 0 h time point. Data are mean ± SEM, n = 8. *p < 0.05, **p < 0.01, ***p < 0.001 compared to time 0.

Studies using cell culture and animal models have suggested that LRRK2 kinase inhibitors may promote the proteosomal degradation of LRRK2 protein^25^. Thus we investigated the effect of inhibitor treatment on total levels of LRRK2 in primary human PBMCs obtained from healthy blood donors over a 24 h time course. All inhibitors induced a significant loss of LRRK2 phosphorylation at Ser935 within the first 2 h time point, and phosphorylation remained diminished over the 24 h time course (Fig. 2). Levels of total LRRK2 were stable over most of the time course, however for all three inhibitors there was a 30–40% reduction in total LRRK2 protein by the 24 h time point. This reduction was not seen with the DMSO treated cells (Fig. 2A). In the case of GSK2578215A and LRRK2-IN1 the decrease at 24 h was statistically significant. These results suggest that continued exposure to LRRK2 inhibitors can reduce LRRK2 protein levels in peripheral human immune cells.

### Similar reductions in LRRK2 phosphorylation with inhibitor treatment in control and PD patient PBMCs

**Figure 3:**
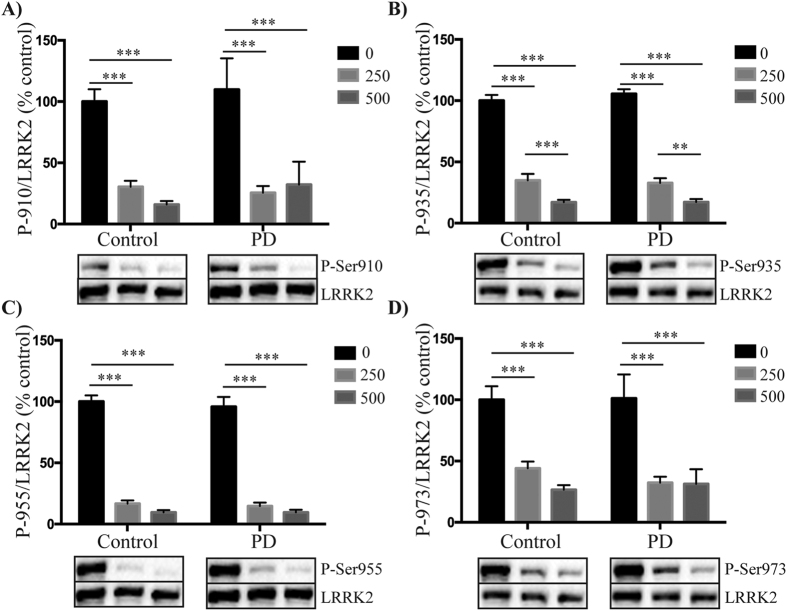
The effect of higher concentrations of GSK2578215A on LRRK2 dephosphorylation in control and PD patient PBMCs. Peripheral blood mononuclear cells isolated from control and PD patients were treated with either 0.5 μM or 0.25 μM GSK2578215A for 1 h. Resulting cell lysates were immunoblotted for LRRK2 and LRRK2 phosphorylated at Ser910 **(A)**, Ser935 **(B)**, Ser955 **(C)** and Ser973 **(D)**. Following quantitation, phosphorylated LRRK2 was normalized to total LRRK2 and results expressed as the percentage reduction in phosphorylation compared to the non-inhibitor treated control group. Two-way ANOVA with Tukey’s post hoc test was used to determine any significant effects, defined as p < 0.05, of inhibitor or disease status on levels of phosphorylation. Data are mean ± SEM. ***p <0.001, **p < 0.01. Representative cropped immunoblots are shown. Fuller western blots are available in Supplementary Figure 1. Sample size is 13 control and 15 PD patients.

**Figure 4:**
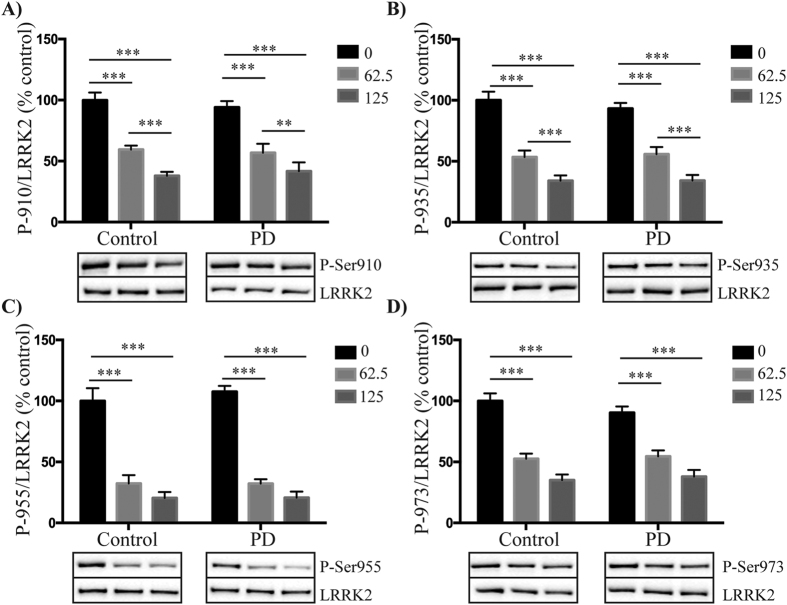
The effect of lower concentrations of GSK2578215A on LRRK2 dephosphorylation in control and PD patient PBMCs. Peripheral blood mononuclear cells isolated from control and PD patients were treated with either 0.125 μM or 0.063 μM GSK2578215A for 1 h. Resulting cell lysates were immunoblotted for LRRK2 and LRRK2 phosphorylated at Ser910 **(A)**, Ser935 **(B)**, Ser955 **(C)** and Ser973 **(D)**. Following quantitation, phosphorylated LRRK2 was normalized to total LRRK2 and results expressed as the percentage reduction in phosphorylation compared to the non-inhibitor treated control group. Two-way ANOVA with Tukey’s post hoc test was used to determine any significant effects, defined as p < 0.05, of inhibitor or disease status on levels of phosphorylation. Data are mean ± SEM. ***p < 0.001, **p < 0.01. Representative cropped immunoblots are shown. Fuller western blots are available in Supplementary Figure 1. Sample size is 12 control and 12 PD patients.

We next assessed whether LRRK2 inhibitors reduced phosphorylation of Ser910, Ser935, Ser955 and Ser973 to a similar extent in PBMCs obtained from PD patients and healthy matched controls. Due to limited patient material only two inhibitors were assessed, GSK2578215A and LRRK2-IN1. Based on previous dose-response experiments in immortalized cell lines^15^,^21^ we initially used the inhibitors at concentrations of 0.5 and 0.25 μM for GSK2578215A, and 1 and 0.5 μM for LRRK2-IN1. At these concentrations, both the GSK2578215A (Fig. 3) and LRRK2-IN1 (Supplementary Figure 2) inhibitors reduced LRRK2 phosphorylation to similar extents in both the control and PD patient PBMCs for all four phosphorylation sites. With the GSK2578215A inhibitor, the least variance was obtained with the P-Ser935 site (Fig. 3B), whilst under the same conditions the P-Ser955 site appeared to be a more sensitive readout (Fig. 3C) and P-Ser973 the least sensitive readout, in terms of percentage reduction in LRRK2 phosphorylation with inhibitor treatment (Fig. 3D). For the LRRK2-IN1 inhibitor, maximal dephosphorylation of all four sites was achieved with the 0.5 μM concentration. To determine if PD patient PBMCs continued to respond similarly to control PBMCs with lower doses of LRRK2 inhibitors, we repeated the experiments in a second cohort of subjects, this time using GSK2578215A at 0.125 and 0.063 μM and LRRK2-IN1 at 0.25 and 0.125 μM. Again, both GSK2578215A (Fig. 4) and LRRK2-IN1 (Supplementary Figure 3) reduced LRRK2 phosphorylation to a similar extent in both the PD and control subject PBMCs at all four phosphorylation sites. These results show that at least at the concentrations employed, LRRK2 kinase inhibitors will work equally well to reduce LRRK2 phosphorylation in primary immune cells from control and PD patients. Thus, monitoring the phosphorylation status of these residues may have utility as a pharmacodynamic biomarker in clinical trials.

## Discussion

Recent years have seen a number of potent and selective inhibitors of LRRK2 kinase activity developed, including by major pharmaceutical and biotechnology companies. The development of these potential drugs has been driven by discoveries that PD-causing missense mutations in LRRK2, especially the relatively common G2019S mutation, increase the enzyme’s kinase activity^10^. Some early studies using pre-clinical models of LRRK2-associated PD also support the concept that LRRK2 kinase inhibition may be of therapeutic benefit^26^,^27^,^28^,^29^. PD caused by the activating LRRK2 G2019S mutation is largely indistinguishable from idiopathic PD^6^. Moreover, genome-wide association studies also link LRRK2 variants to idiopathic PD^1^,^2^,^3^. Thus, although it remains to be established whether LRRK2 kinase activity is increased in idiopathic PD, there is a possibility that LRRK2 kinase inhibitors may also be of benefit for this more common form of PD.

In the event that clinical trials of LRRK2 kinase inhibitors are ultimately warranted, it is important to have robust readouts of inhibitor target engagement. The most commonly used readout of LRRK2 target engagement involves monitoring the loss of phosphorylation on a cluster of serine residues located near the N-terminus of LRRK2, particularly Ser935, which occurs with inhibitor treatment^13^. However, LRRK2 kinase activity is not the sole regulator of phosphorylation at these residues, rather it is a yet to be fully elucidated result of a complex interplay between LRRK2 and other upstream kinases and phosphatases^13^,^17^,^18^,^19^,^30^. It is therefore important to establish whether LRRK2 inhibitor-induced reductions in serine phosphorylation still occur in cells from PD patients, and if so is it similar in extent to controls.

In this study we have therefore examined PBMCs from control and PD subjects. It is established that LRRK2 is highly expressed in PBMCs, particularly monocytes and B-lymphocytes^31^, providing a convenient source of peripheral primary cells. It has also been demonstrated that levels of LRRK2 and its phosphorylation at Ser910 and Ser935 are unaltered in idiopathic PD PBMCs^32^. Moreover, the levels were not influenced by disease duration, disease severity, levodopa medication or age, suggesting that LRRK2 in PBMCs is stable and suitable for use as a pharmacodynamic biomarker^32^. Our new data using *ex vivo* treatment of PBMCs from control and PD patients now demonstrates that inhibitor-induced dephosphorylation of Ser910, Ser935, Ser955 and Ser973 is also similar between control and PD patients. This suggests that the complex pathways regulating LRRK2 serine phosphorylation are unperturbed in idiopathic PD PBMCs, and adds further validity to the use of these residues as peripheral pharmacodynamic readouts.

This study employed concentrations of inhibitors previously shown to induce a dose-dependent reduction in LRRK2 phosphorylation in model cell systems^15^,^21^. In the primary human PBMCs however, these same concentrations caused near maximal loss of phosphorylation, suggesting these cells may be more sensitive to LRRK2 inhibitors. Even when lower concentrations of inhibitors were used we still did not find a significant difference between control and PD patient cells however, it may be prudent to perform IC50 curves with actual clinical trial drugs once these have been established and a better idea of the therapeutic window of LRRK2 drugs is known. Moreover, validation using higher throughput readouts of LRRK2 phosphorylation, such a LRRK2 P-Ser935 ELISA assay^33^, would be important for larger scale studies. Our results also suggest a mild effect of LRRK2 inhibitors at high concentrations for 24 h to reduce total LRRK2 levels, as has been noted in cell culture studies^25^. It may thus also be important to monitor total LRRK2, particularly if treatments are extended over days. It is also noteworthy that the highest inhibitor concentrations used were well tolerated by the cells with no acute toxicity observed.

In summary we provide evidence that reduced phosphorylation of LRRK2 on residues Ser910, Ser935, Ser955 and/or Ser973 can be used as a measure of peripheral target engagement for LRRK2 inhibitors in primary human cells from PD patients.

## Additional Information

**How to cite this article**: Perera, G. *et al*. Inhibitor treatment of peripheral mononuclear cells from Parkinson’s disease patients further validates LRRK2 dephosphorylation as a pharmacodynamic biomarker. *Sci. Rep*. **6**, 31391; doi: 10.1038/srep31391 (2016).

## Supplementary Material

Supplementary Information

## Figures and Tables

**Table 1:** Subject demographics.

Study 1	Control	Parkinson’s Disease
Number	13	15
Age (years)	67 ± 1.4	68 ± 1.5
Gender	4M/10F	11M/4F
Disease Duration (months)	—	37 ± 8
Levodopa equivalent dose (mg)	—	707 ± 35
MDS UPDRS (total score)	—	20 ± 3
**Study 2**	**Control**	**Parkinson’s Disease**
Number	12	12
Age (years)	68 ± 2.6	64 ± 2.7
Gender	8M/4F	10M/2F
Disease Duration (months)	—	65 ± 13
Levodopa equivalent dose (mg)	—	735 ± 58
MDS UPDRS (total score)	—	22 ± 2.5

Demographic data for subjects involved in this study. MDS-UPDRS = Movement Disorder Society Unified Parkinson’s Disease Rating Scale. Data are mean ± SEM.

## References

[b1] Nalls M. A. . Large-scale meta-analysis of genome-wide association data identifies six new risk loci for Parkinson’s disease. Nature genetics 46, 989–993 (2014). 25064009 10.1038/ng.3043 PMC4146673

[b2] Simon-Sanchez J. . Genome-wide association study reveals genetic risk underlying Parkinson’s disease. Nature genetics 41, 1308–1312 (2009). 19915575 10.1038/ng.487 PMC2787725

[b3] Satake W. . Genome-wide association study identifies common variants at four loci as genetic risk factors for Parkinson’s disease. Nature genetics 41, 1303–1307 (2009). 19915576 10.1038/ng.485

[b4] Zimprich A. . Mutations in LRRK2 cause autosomal-dominant parkinsonism with pleomorphic pathology. Neuron 44, 601–607 (2004). 15541309 10.1016/j.neuron.2004.11.005

[b5] Paisan-Ruiz C. . Cloning of the gene containing mutations that cause PARK8-linked Parkinson’s disease. Neuron 44, 595–600 (2004). 15541308 10.1016/j.neuron.2004.10.023

[b6] Healy D. G. . Phenotype, genotype, and worldwide genetic penetrance of LRRK2-associated Parkinson’s disease: a case-control study. Lancet neurology 7, 583–590 (2008). 18539534 10.1016/S1474-4422(08)70117-0 PMC2832754

[b7] Gilligan P. J. Inhibitors of leucine-rich repeat kinase 2 (LRRK2): progress and promise for the treatment of Parkinson’s disease. Curr Top Med Chem 15, 927–938 (2015). 25832719 10.2174/156802661510150328223655

[b8] West A. B. Ten years and counting: moving leucine-rich repeat kinase 2 inhibitors to the clinic. Movement disorders 30, 180–189 (2015). 25448543 10.1002/mds.26075 PMC4318704

[b9] Martin I., Kim J. W., Dawson V. L. & Dawson T. M. LRRK2 pathobiology in Parkinson’s disease. Journal of neurochemistry 131, 554–565 (2014). 25251388 10.1111/jnc.12949 PMC4237709

[b10] Jaleel M. . LRRK2 phosphorylates moesin at threonine-558: characterization of how Parkinson’s disease mutants affect kinase activity. The Biochemical journal 405, 307–317 (2007). 17447891 10.1042/BJ20070209 PMC1904520

[b11] Deng X., Choi H. G., Buhrlage S. J. & Gray N. S. Leucine-rich repeat kinase 2 inhibitors: a patent review (2006–2011). Expert opinion on therapeutic patents 22, 1415–1426 (2012). 23126385 10.1517/13543776.2012.729041

[b12] Kethiri R. R. & Bakthavatchalam R. Leucine-rich repeat kinase 2 inhibitors: a review of recent patents (2011–2013). Expert opinion on therapeutic patents 24, 745–757 (2014). 24918198 10.1517/13543776.2014.907275

[b13] Dzamko N. . Inhibition of LRRK2 kinase activity leads to dephosphorylation of Ser(910)/Ser(935), disruption of 14-3-3 binding and altered cytoplasmic localization. The Biochemical journal 430, 405–413 (2010). 20659021 10.1042/BJ20100784 PMC3631100

[b14] Nichols R. J. . 14-3-3 binding to LRRK2 is disrupted by multiple Parkinson’s disease-associated mutations and regulates cytoplasmic localization. The Biochemical journal 430, 393–404 (2010). 20642453 10.1042/BJ20100483 PMC2932554

[b15] Deng X. . Characterization of a selective inhibitor of the Parkinson’s disease kinase LRRK2. Nature chemical biology 7, 203–205 (2011). 21378983 10.1038/nchembio.538 PMC3287420

[b16] Doggett E. A., Zhao J., Mork C. N., Hu D. & Nichols R. J. Phosphorylation of LRRK2 serines 955 and 973 is disrupted by Parkinson’s disease mutations and LRRK2 pharmacological inhibition. Journal of neurochemistry 120, 37–45 (2012). 22004453 10.1111/j.1471-4159.2011.07537.x

[b17] Dzamko N. . The IkappaB kinase family phosphorylates the Parkinson’s disease kinase LRRK2 at Ser935 and Ser910 during Toll-like receptor signaling. PloS one 7, e39132 (2012). 22723946 10.1371/journal.pone.0039132 PMC3377608

[b18] Lobbestael E. . Identification of protein phosphatase 1 as a regulator of the LRRK2 phosphorylation cycle. The Biochemical journal 456, 119–128 (2013). 23937259 10.1042/BJ20121772 PMC5141581

[b19] Chia R. . Phosphorylation of LRRK2 by casein kinase 1alpha regulates trans-Golgi clustering via differential interaction with ARHGEF7. Nature communications 5, 5827 (2014). 10.1038/ncomms6827 PMC4268884 25500533

[b20] Henderson J. L. . Discovery and preclinical profiling of 3-[4-(morpholin-4-yl)-7H-pyrrolo[2,3-d]pyrimidin-5-yl]benzonitrile (PF-06447475), a highly potent, selective, brain penetrant, and *in vivo* active LRRK2 kinase inhibitor. Journal of medicinal chemistry, 58, 419–132 (2015). 25353650 10.1021/jm5014055

[b21] Reith A. D. . GSK2578215A; a potent and highly selective 2-arylmethyloxy-5-substitutent-N-arylbenzamide LRRK2 kinase inhibitor. Bioorganic & medicinal chemistry letters 22, 5625–5629 (2012). 22863203 10.1016/j.bmcl.2012.06.104 PMC4208292

[b22] Goetz C. G. . Movement Disorder Society-sponsored revision of the Unified Parkinson’s Disease Rating Scale (MDS-UPDRS): scale presentation and clinimetric testing results. Movement disorders 23, 2129–2170 (2008). 19025984 10.1002/mds.22340

[b23] Tomlinson C. L. . Systematic review of levodopa dose equivalency reporting in Parkinson’s disease. Movement disorders 25, 2649–2653 (2010). 21069833 10.1002/mds.23429

[b24] Davies P. . Comprehensive characterization and optimization of anti-LRRK2 (leucine-rich repeat kinase 2) monoclonal antibodies. The Biochemical journal 453, 101–113 (2013). 23560750 10.1042/BJ20121742 PMC3682752

[b25] Zhao J., Molitor T. P., Langston J. W. & Nichols R. J. LRRK2 dephosphorylation increases its ubiquitination. The Biochemical journal 469, 107–120 (2015). 25939886 10.1042/BJ20141305 PMC4613513

[b26] Daher J. P. . Leucine-rich Repeat Kinase 2 (LRRK2) Pharmacological Inhibition Abates alpha-Synuclein Gene-induced Neurodegeneration. The Journal of biological chemistry 290, 19433–19444 (2015). 26078453 10.1074/jbc.M115.660001 PMC4528108

[b27] Liu Z. . Inhibitors of LRRK2 kinase attenuate neurodegeneration and Parkinson-like phenotypes in Caenorhabditis elegans and Drosophila Parkinson’s disease models. Human molecular genetics 20, 3933–3942 (2011). 21768216 10.1093/hmg/ddr312 PMC3177653

[b28] Lee B. D. . Inhibitors of leucine-rich repeat kinase-2 protect against models of Parkinson’s disease. Nature medicine 16, 998–1000 (2010). 10.1038/nm.2199 PMC2935926 20729864

[b29] Yao C. . Kinase inhibitors arrest neurodegeneration in cell and C. elegans models of LRRK2 toxicity. Human molecular genetics 22, 328–344 (2013). 23065705 10.1093/hmg/dds431 PMC3526163

[b30] Li X. . Phosphorylation-dependent 14-3-3 binding to LRRK2 is impaired by common mutations of familial Parkinson’s disease. PloS one 6, e17153 (2011). 21390248 10.1371/journal.pone.0017153 PMC3046972

[b31] Hakimi M. . Parkinson’s disease-linked LRRK2 is expressed in circulating and tissue immune cells and upregulated following recognition of microbial structures. Journal of Neural Transmission 118, 795–808 (2011). 21552986 10.1007/s00702-011-0653-2 PMC3376651

[b32] Dzamko N., Chua G., Ranola M., Rowe D. B. & Halliday G. M. Measurement of LRRK2 and Ser910/935 phosphorylated LRRK2 in peripheral blood mononuclear cells from idiopathic Parkinson’s disease patients. Journal of Parkinson’s disease 3, 145–152 (2013). 10.3233/JPD-130174 23938344

[b33] Delbroek L. . Development of an enzyme-linked immunosorbent assay for detection of cellular and *in vivo* LRRK2 S935 phosphorylation. J Pharm Biomed Anal 76, 49–58 (2013). 23313773 10.1016/j.jpba.2012.12.002 PMC4196644

