# Supplementary material for: Inhibitor treatment of peripheral mononuclear cells from Parkinson’s disease patients further validates LRRK2 dephosphorylation as a pharmacodynamic biomarker
Source: Sci Rep. 2016 Aug 9;6:31391. doi: 10.1038/srep31391 (PMC4977566; doi:10.1038/srep31391)
Supplement: Supplementary Information [file srep31391-s1.pdf]

Inhibitor treatment of peripheral mononuclear cells from Parkinson's disease patients further validates LRRK2 dephosphorylation as a pharmacodynamic biomarker.

Perera G[1], Ranola M[2], Rowe D.B[2], Halliday G.M[1,3], Dzamko N[1,3*]

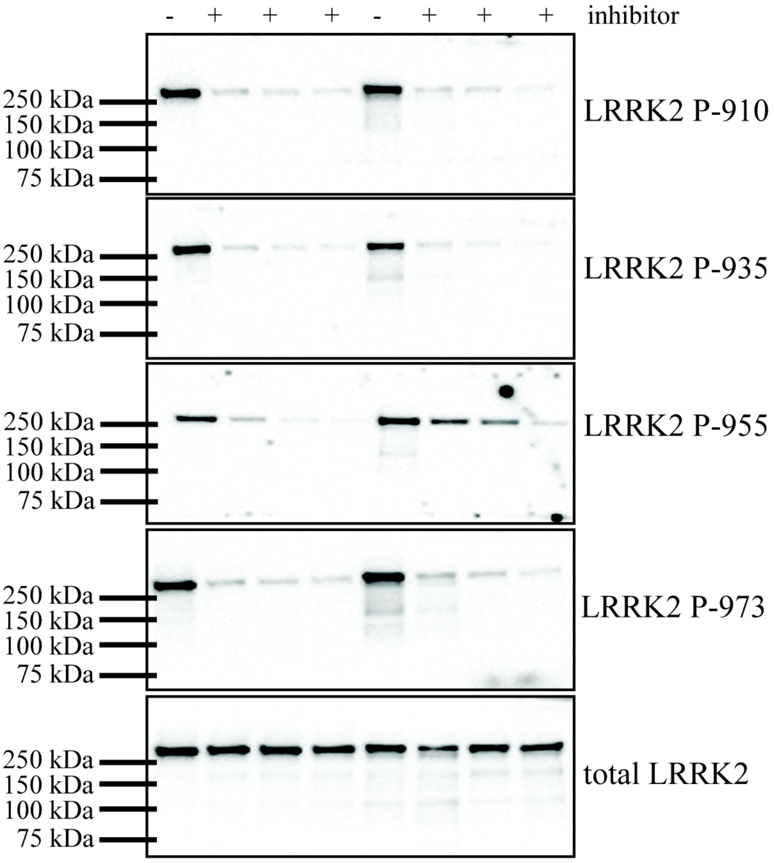

Supplementary Figure 1

Supplementary Figure 1. Immunoblots showing antibody specificity. Example full western blots from two subjects treated +/- GSK2578215A as used to generate data for figure 3. The antibodies, which have been well validated in knockout cells, detect a single band at the correct size of LRRK2. Note that membranes were cut at below the 75 kDa marker for simultaneous immunoblot detection of beta-actin at 50 kDa.

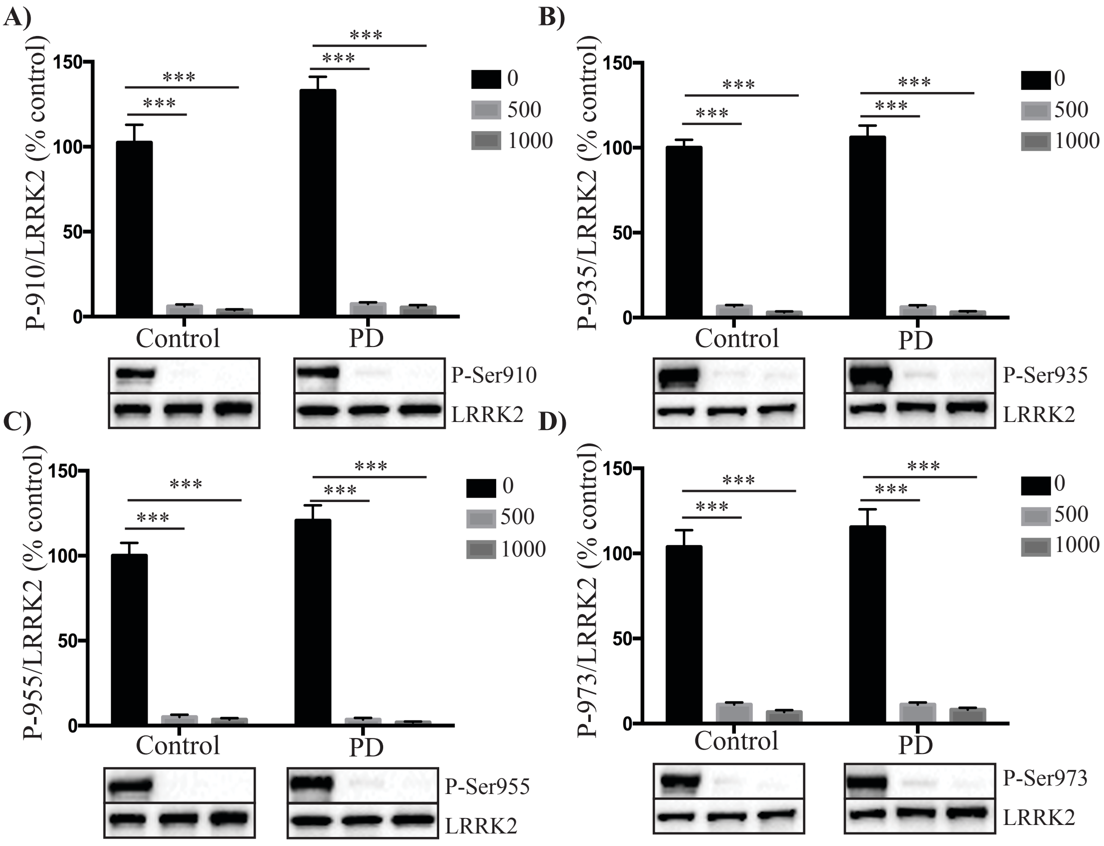

Supplementary Figure 2

Supplementary Figure 2. The effect of higher concentrations of LRRK2-IN1 on LRRK2 dephosphorylation in control and PD patient PBMCs. PBMCs isolated from control and PD patients were treated with either 0.5 μM or 1 μM LRRK2-IN1 for 1 h. Resulting cell lysates were immunoblotted for LRRK2 and LRRK2 phosphorylated at Ser910 (**A**), Ser935 (**B**), Ser955 (**C**) and Ser973 (**D**). Following quantitation, phosphorylated LRRK2 was normalized to total LRRK2 and results expressed as the percentage reduction in phosphorylation compared to the non-inhibitor treated control group. Two-way ANOVA with Tukey's post hoc test was used to determine any significant effects, defined as $p < 0.05$, of inhibitor or disease status on levels of phosphorylation. Data are mean ± SEM. *** = $p < 0.001$, ** = $p < 0.01$. Representative immunoblots are shown. Sample size is 13 control and 15 PD patients.

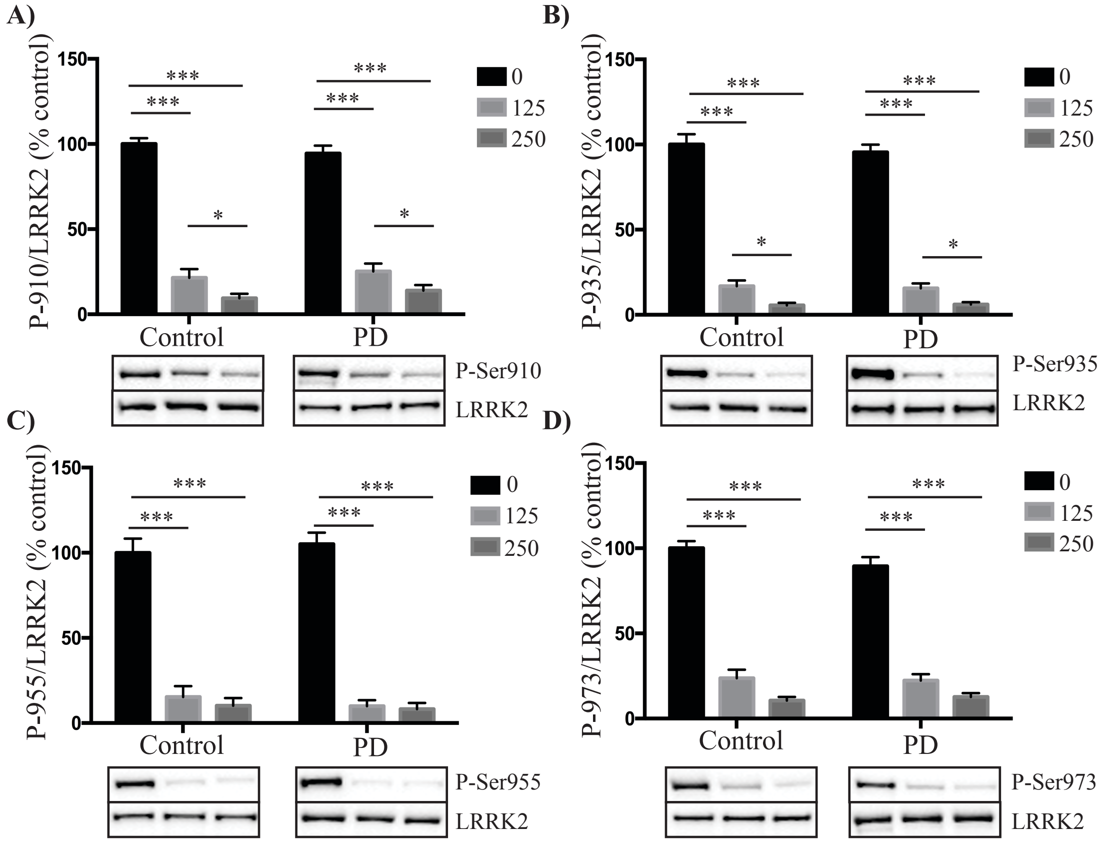

Supplementary Figure 3

Supplementary Figure 3. The effect of lower concentrations of LRRK2-IN1 on LRRK2 dephosphorylation in control and PD patient PBMCs. PBMCs isolated from control and PD patients were treated with either 0.5 µM or 1 µM LRRK2-IN1 for 1 h. Resulting cell lysates were immunoblotted for LRRK2 and LRRK2 phosphorylated at Ser910 (**A**), Ser935 (**B**), Ser955 (**C**) and Ser973 (**D**). Following quantitation, phosphorylated LRRK2 was normalized to total LRRK2 and results expressed as the percentage reduction in phosphorylation compared to the non-inhibitor treated control group. Two-way ANOVA with Tukey's post hoc test was used to determine any significant effects, defined as $p < 0.05$, of inhibitor or disease status on levels of phosphorylation. Data are mean ± SEM. *** = $p< 0.001$, ** = $p< 0.01$, * = $p < 0.05$. Representative immunoblots are shown. Sample size is 12 control and 12 PD patients.
